# Overcoming barriers to prostate cancer care in the Philippines

**DOI:** 10.3389/fruro.2023.1336179

**Published:** 2024-02-26

**Authors:** Thomas Vincent Vergara, Juan Martin Magsanoc, Marvin Jonne Mendoza, Gerardo Tomas Cornelio, Rudolfo De Guzman, Nikko Magsanoc, Dennis Serrano

**Affiliations:** ^1^ St. Luke’s Medical Center – Global City, Department of Radiation Oncology, Taguig City, Philippines; ^2^ St. Luke’s Medical Center – Global City, Cancer Institute, Taguig City, Philippines; ^3^ Department of Internal Medicine, Section of Medical Oncology, National Kidney & Transplant Institute, Quezon City, Philippines; ^4^ St. Luke’s Medical Center – Global City, Institute of Urology, Taguig City, Philippines; ^5^ Department of Urology, National Kidney & Transplant Institute, Quezon City, Philippines

**Keywords:** prostate cancer, global health, Philippines, barriers, health equity

## Abstract

Prostate cancer (PCa) is a health concern affecting millions of men globally, with a concerning rise in incidence in the Philippines, a country that faces a complex set of barriers to equitable access to quality PCa care. In this article, we describe the unique geographic, economic, socio-cultural, and political factors that influence access to screening, diagnosis, treatment, and supportive services in the country, and explore avenues for development. The country lacks a nationwide PCa registry to inform resource allocation and guide PCa cancer care programs and policy. Misconceptions, cultural barriers and negative attitudes about PCa among Filipino men adversely influence health-seeking behavior. Inadequate insurance coverage, and high out-of-pocket costs obstruct access to essential care. Geographic and political factors contribute to the uneven distribution of healthcare resources needed for comprehensive PCa care, including access to medical specialists, essential medicines, and surgical and radiotherapeutic equipment. Overcoming these challenges requires a collaborative effort encompassing robust data collection, awareness campaigns to reshape societal norms, policy and economic reforms, infrastructure improvements, and enhanced collaboration among healthcare professionals to provide evidence-based care. Addressing these issues holistically can pave the way to better outcomes and improved quality of life for Filipino men with this life-altering disease.

## 1 Introduction

Prostate cancer (PCa) is a global health concern affecting millions of men worldwide. An analysis of GLOBOCAN 2020 estimates that between the years 2000 and 2019, the incidence of PCa in the Philippines increased at an average of 0.58% annually (1). The Philippine Cancer Society reported that PCa incidence in two urbanized regions in the country had increased at a rate of 2.1% per year between 1980 and 2007 (2). In 2020, it is estimated that 8242 Filipino men were newly diagnosed, making PCa the third most incident cancer in males, and the fifth most common overall (3). Despite being more prevalent in developed countries, PCa age-standardized mortality rates are disproportionately higher in developing countries such as the Philippines (1). A systematic review on PCa survival rates in Asian countries demonstrated that the Philippines had the lowest 5-year survival rate in the region (4).

The Philippines is a Southeast Asian nation-archipelago comprising of 111 million individuals in 2022, with a GDP per capita of USD 3,498.5. Nearly half (46%) of the population reside in rural areas, and almost 80% identify as Roman Catholic.

PCa care in the Philippines presents a unique set of challenges, including limited access to screening, diagnosis, treatment, and supportive care services which are all influenced by the interaction of complex geographic, socio-cultural, economic, and political factors. In this article, we will explore the different challenges that limit effective and equitable access to medical care among Filipino men with PCa.

## 2 Establishing a comprehensive PCa cancer registry

The absence of a robust nationwide cancer registry makes the precise epidemiology of PCa in the country difficult to establish. Philippine PCa incidence figures reported in GLOBOCAN 2020 are extrapolated using prediction models from the 2017 average of three subnational registries with limited catchment areas (3, 5). Urban-rural disparities in incidence and outcomes may not be adequately reflected by data from registries restricted largely to urban/semi-urban regions. Efforts have been undertaken to estimate PCa epidemiology based on nationwide administrative data on the utilization of healthcare services from the National Health Insurance Program (NHIP), but have been fraught by inherent limitations (5). Limited access to screening and testing in rural areas can likewise influence epidemiologic reporting. Accurate historical PCa incidence data for the entire country simply does not exist, and there is no local data on the distribution of PCa according to stage or risk stratification at the time of diagnosis.

The National Integrated Cancer Control Act (NICCA) of 2019 mandated the establishment of a population-based national cancer registry to provide a framework for assessing and controlling the impact of cancer care in various communities (6). Under the law, all hospitals are tasked to create their own hospital-based cancer registries in cooperation with the Department of Health (DOH). However, these provisions of the NICCA have yet to be fully implemented. A private-driven initiative called Cancer CARE Registry Philippines (CARE PH) provides a web-based CARE application that hospitals can utilize to establish their own registries. The CARE app also has the capacity to share anonymized data with a secure and encrypted central database, whose summary data can be accessed through the CARE PH website (7). Such initiatives can be used by PCa care providers to boost data collection for epidemiologic reporting of PCa. Accurate data collection and cancer registries are essential for understanding the prevalence and distribution of PCa among Filipinos in order to guide the allocation of resources, the conduct of research, and the development of targeted interventions.

## 3 Lack of awareness, cultural barriers, and negative attitudes

It has been demonstrated that Filipino men living in Hawaii are more likely to be diagnosed with PCa at a later stage and experience higher mortality rates compared with other ethnic subgroups (8). A survey among this group suggests that misinformation, and lack of awareness and knowledge about PCa contributes to attitudes of fatalism, dread, and hopelessness - all of which lead to poorer health-seeking behavior (9). These are compounded by socio-culturally conditioned perceptions of masculinity. Elderly Filipino males are less likely to report health concerns, injury or sickness compared to females (10). Traditional notions of masculinity in the Philippines are often associated with traits such as stoicism, self-sufficiency, and physical strength. Men are expected to embody these attributes, which can discourage them from seeking medical attention. Expressing vulnerability or acknowledging health concerns is sometimes perceived as a sign of weakness, leading to a reluctance to address health issues promptly, particularly when these concerns are related to sexual function (11).

In addition, beliefs in traditional and alternative medicine such as herbal concoctions and dietary supplements, traditional rituals, and faith-healers are still deeply embedded in poorer and rural communities (12). A local survey among Filipino cancer patients indicates that these non-conventional alternatives can consume up to 50% of patients’ income, despite limited evidence for their efficacy (13).

To overcome these issues, the DOH has designated the month of June of every year as “Prostate Cancer Disease Awareness Month.” During the month, the Philippine Urological Association (PUA) in partnership with the DOH implements programs that aim to train primary care physicians on PCa early detection and screening, to increase lay awareness, and to provide free consultations/tele-consults at institutions specializing in urologic care. Such educational efforts need to be sustained, intensified, and initiated at an earlier age. Teaching young boys that being health-conscious is a sign of strength rather than weakness can shift societal norms over time. In a nation with one of the highest rates of internet usage, social media can be a particularly powerful tool for implementing culturally-relevant information campaigns that aim to dispel myths, and emphasize the significance of early detection for favorable treatment outcomes. Building support networks and providing relatable role models can likewise make a significant impact. Peer discussions and testimonials from men who have sought medical care can encourage others to overcome their hesitations and seek help when needed. In the context of the Philippines, a country where traditions, beliefs, norms, and gender expectations play a significant role, understanding how cultural factors influence men’s approach to healthcare is of paramount importance.

## 4 Financial barriers

Health care financing in the Philippines is provided through a dual healthcare delivery system comprising the public and private sectors. Although government expenditure for health has generally continued to increase since 2005, household out-of-pocket spending still contributed to 44.7% of the Current Health Expenditure (CHE) in 2022, an increase of 5% from the previous year. Inequalities in access to health services related to financing can be gleaned from the fact that health spending of the top 5th income quintile (32.2% of CHE) was nearly equal to that of the lowest 2 quintiles combined (32.6%) in 2022. Cancer-related spending accounted for only 5.5% of CHE in 2022, but is still higher compared to the average of 2.78% between the years 2014 and 2019 (14). Data on national spending for PCa-related services could not be determined, but the cost for various diagnostic and therapeutic procedures will vary between government and private hospitals, and between private institutions.

An analysis of the Filipino cohort in the ACTION Study (which looked at financial catastrophe associated with cancer treatment in South-East Asia) concluded that although having insurance coverage lowered the odds of treatment discontinuation, it did not have a statistically significant impact on financial catastrophe prevention among Filipino cancer patients (15). This is likely due to inadequate benefit packages or support value provided by most insurance schemes, including the NHIP through the Philippine Health Insurance Corporation or PhilHealth. For example, PhilHealth’s Z Benefit Package (launched in 2011 to provide financial risk protection for diseases that are potentially economically catastrophic) only includes low- and intermediate-risk prostate cancer (16). Furthermore, uptake of the package has been limited by lack of awareness among physicians who do not refer eligible patients, and by difficulties in accessing the program for patients not diagnosed in a PhilHealth-accredited hospital which has been contracted to offer the benefit packages. As of 2023, there are only 7 hospitals in three administrative regions that are contracted to offer the Z Benefit Package for PCa (17). Insurance population coverage by PhilHealth has been reported to be as high as 92%, but its share in healthcare expenditure averages only 30% (18). Although it has been tasked to address important issues in national healthcare financing through its role in implementing the Universal Healthcare Act of 2019 (19), the PhilHealth Corporation has in recent years been beset with allegations of financial mismanagement and bureaucratic inefficiency (20).

For many Filipinos, the cost of PCa screenings, diagnostic tests, treatments, and medications can be prohibitive, particularly those with limited financial resources. Out-of-pocket spending, particularly for high-cost procedures related to cancer can deter individuals from seeking necessary medical attention.

## 5 Geographic barriers

Geographic barriers, especially in rural and remote areas, contribute to limited access to specialized cancer care facilities. The Philippines’ archipelagic nature, compounded by inadequate transportation infrastructure, makes it challenging for patients to reach treatment centers in a timely manner. Travel costs, accommodation expenses, and loss of income due to treatment-related travel can be burdensome for patients and their families.

Healthcare resources, including medical personnel, equipment, and funding, are concentrated in urban areas, leaving rural and underserved regions with severely limited access to advanced cancer treatment options. For example, in 2022, 26% of CHE was spent in the National Capital Region (NCR), although its residents comprise only 12% of the entire country’s population (14). The devolution of the national government’s healthcare services to local government units (LGUs), although aimed at empowering local communities in addressing their specific needs, has likely contributed to greater unevenness in the quality and accessibility of services, and has made the healthcare sector vulnerable to the politicization of decision making in resource management and service delivery (21). Full implementation of the NICCA, including its provisions on the establishment of regional cancer care hospitals throughout the country can help alleviate wide disparities in the accessibility and quality of PCa care. In the meantime, a streamlined referral network between rural healthcare services and facilities with adequate capabilities for PCa treatment needs to be established.

## 6 Diagnostics

There is limited up-to-date information on the accessibility of essential diagnostic tests for PCa patients in the Philippines. Although most facilities are capable of Prostate Specific Antigen (PSA) testing, information on the adequacy of imaging equipment is lacking. In 2013, there were only 1.09 Computed Tomography (CT) machines and 0.3 Magnetic Resonance Imaging (MRI) equipment for every 1 million Filipinos, lagging behind its neighboring countries (22). There is no data on the accessibility of nuclear imaging, including Bone Scintigraphy and Positron Emission Tomography (PET), including Prostate-Specific Membrane Antigen PET (PSMA PET). Genetic testing and molecular/biomarker analysis in PCa, although increasingly relevant in the era of personalized medicine, are not readily available and often require sending out specimens to neighboring countries. Issues with accessibility of testing are compounded by variability of coverage under the NHIP, leading to high out-of-pocket spending for diagnostic procedures (23).

## 7 Treatment

In urban areas, it is not uncommon for patients to consult directly with a urologist after developing genitourinary symptoms. In poorer rural areas, they are more likely to be seen by a primary care physician or local health worker who refers them to the appropriate specialist. The urologist typically performs the initial assessment and diagnosis, and discusses the available treatment options with the patient. No data is available on the referral rates to multidisciplinary team discussions (MDTs) for PCa in the Philippines, but its utilization depends largely on widely variable factors including accessibility of MDTs, institutional policy, and urologist initiative. This is certainly an area that requires a collaborative effort involving healthcare professionals, institutions, policymakers, and stakeholders. MDTs play a crucial role in providing comprehensive and coordinated care for PCa patients, by helping establish more accurate and complete preoperative staging, providing guidance on the appropriate use of diagnostic tests, and limiting unnecessary, futile, or even harmful treatments (24). In order to provide evidence-based recommendations for clinicians, the National Kidney Transplant Institute (NKTI), under the direction of the DOH and in accordance with the provisions of the NICCA, published the 1st edition of the Philippine Clinical Practice Guideline for the Diagnosis and Management of Prostate Cancer in 2021 (25).

When considering potential curative treatment options, patients may view less invasive approaches such as radiotherapy (RT) as less favorable when compared to surgical procedures; and urologists who are unfamiliar with the nuances of RT might be less likely to recommend it, even in situations when outcomes are comparable. The choice between radical prostatectomy and RT for definitive treatment of PCa is not only influenced by patient and physician preferences, but also depends on the cost of procedures and ease of access to RT facilities, of which about 40% of are located in or around the NCR. Four of the 17 administrative regions that make up the country do not have a single RT facility or in-house radiation oncologist (25). Due to high public demand, it is not unusual for RT to be delayed for months in public facilities. The use of shorter courses of RT (hypofractionation) in the treatment of PCa is increasing and is encouraged, as it directly reduces costs and offers significant logistical benefits for both patients and RT providers. Although there are no cost-effectiveness studies that directly compare the two interventions, both entail significant cost, with radiotherapy likely incurring lower direct costs than radical prostatectomy (26). Access to surgery and the availability of surgical specialists are also critical areas that need further investigation and development. Even the indirect costs associated with RT and/or surgical treatment (travel, accommodation, loss of livelihood) can be significant deterrents for patients with PCa from seeking treatment once they are diagnosed.

Hormonal and systemic agents for advanced or metastatic prostate cancer in the Philippine National Drug Formulary include goserelin, leuprolide, flutamide, bicalutamide, cyproterone, docetaxel, and mitoxantrone, but does not include abiraterone, immunotherapy and targeted agents (27). Access to these, however, can still be difficult, particularly in rural areas where health delivery systems are inadequate, and costs can be prohibitive, especially with long-term use (28). Consistency in the supply and availability of these drugs in rural areas can be a significant issue. Surgical castration and medical castration are equally effective forms of androgen deprivation therapy (ADT) but the former is probably underutilized despite its cost-effectiveness (29). Whenever ADT is necessary, long-term costs should be discussed, and patients who are likely to prematurely discontinue medical ADT due to financial constraints can be offered surgical castration as an option.

The attitudes of Filipino men and their health care providers toward what can be perceived as “less active” approached after PCa diagnosis need to be explored. Observation and active surveillance are equally safe and effective in selected patients with low-risk PCa or those who have shorter life expectancy. However, it has been our experience that many Filipino men with PCa can have an aversion to such approaches, which may be due to misconceptions about the varied clinical trajectories of PCa. Such attitudes can be influenced by fear of disease progression, misunderstanding of the different risk levels and trajectories of PCa, psychological anxiety related to a cancer diagnosis, and the influence of peers and family. It is therefore not uncommon for patients to switch to physicians who are willing to perform what they regard as necessary interventions after the diagnosis is made. In addition, factors that influence a physician’s decision to offer a more comprehensive set of options to patients (i.e. active surveillance, observation, or non-surgical approaches) have to be studied. These may include orientations and biases related to their own specialization, historical practices, and concerns about liability.

## 8 Conclusion

In conclusion, the challenges surrounding PCa care in the Philippines are multifaceted and require a comprehensive approach for effective resolution. The absence of a comprehensive national cancer registry hinders our ability to fully understand the epidemiological landscape of PCa in the country. Cultural barriers and negative attitudes among Filipino men highlight the need for ongoing awareness campaigns and educational initiatives that aim to reshape societal norms, perceptions of masculinity, and health-seeking behaviors. Financial barriers, both in terms of limited insurance coverage and high out-of-pocket spending, continue to impede access to essential care, and require effective economic reforms and strong political will. Geographic barriers, particularly in rural areas necessitate investments in transportation infrastructure and the equitable distribution of healthcare resources. Furthermore, enhancing collaboration among healthcare professionals is vital for promoting MDTs and facilitating the adoption of evidence-based treatment approaches. In this intricate web of challenges, a collective effort is required to overcome the barriers and disparities that currently limit access to quality prostate cancer care in the Philippines. By addressing these issues comprehensively, we can aspire to improve the prognosis and quality of life for Filipino men affected by this life-altering illness.

## Data availability statement

The original contributions presented in the study are included in the article/supplementary material. Further inquiries can be directed to the corresponding author.

## Author contributions

TV: Writing – original draft. JM: Conceptualization, Writing – original draft, Writing – review & editing. MM: Writing – review & editing. GC: Writing – review & editing. RDG: Writing – review & editing. NM: Writing – review & editing. DS: Writing – review & editing.
